# A first-in-human phase I study to evaluate the safety, tolerability, and pharmacokinetics of a novel anti-influenza agent suraxavir marboxil in healthy Chinese subjects

**DOI:** 10.1128/aac.00685-25

**Published:** 2025-09-30

**Authors:** Qingqing Wu, Lang Lv, Shousheng Yan, Yijun Wang, Qian Chen, Wenjing Xu, Yun Liu, Wei Wang, Jingying Jia, Chen Yu, Jingjing Chen, Yanmei Liu

**Affiliations:** 1 Drug Clinical Trial Center, Shanghai Xuhui Central Hospital/Xuhui Hospital, Fudan University, Shanghai, Shanghai, China; 2 Shanghai Engineering Research Center of Phase I Clinical Research & Quality Consistency Evaluation for Drugs, Shanghai, China; 3 Jiangxi Qingfeng Pharmaceutical Co., Ltd., Jiangxi, China; Providence Portland Medical Center, Portland, Oregon, USA

**Keywords:** influenza, suraxavir marboxil, polymerase acidic protein inhibitor, safety, pharmacokinetics, healthy subjects

## Abstract

Suraxavir marboxil (GP681) is a prodrug of a novel polymerase acidic protein inhibitor, and its metabolite GP1707D07 prevents the replication of influenza virus by selectively inhibiting the cap-dependent nucleic acid endonuclease of influenza virus. This study evaluates the safety, tolerability, and pharmacokinetics of suraxavir marboxil after a single dose and assesses the effect of a high-fat, high-calorie meal on the pharmacokinetics of suraxavir marboxil in healthy Chinese subjects. The study included two parts: single ascending-dose study (SAD) and food effect study (FE). In SAD, subjects were randomized to single-dose suraxavir marboxil (20, 40, 60, or 80 mg) or placebo. In FE, subjects (*n* = 16) were randomized to single-dose suraxavir marboxil 40 mg in fasting and fed states. Safety assessment and sample collection were in accordance with the protocol. Suraxavir marboxil was well tolerated in healthy Chinese subjects in both SAD and FE, and all adverse events recovered without treatment after discontinuation of suraxavir marboxil. In SAD, after administration of suraxavir marboxil in the dosage range of 20–80 mg, the time to maintain the clinically defined effective target blood concentration is about 72–136 h. In FE, a high-fat, high-calorie meal reduced *C*_max_ by approximately 19% and AUC_0–∞_ by approximately 15%. Suraxavir marboxil was well tolerated in healthy Chinese subjects. Based on the safety and pharmacokinetic data, 20–80 mg single oral dosing was supported for further clinical development. Food intake may slightly reduce the rate and extent of absorption of suraxavir marboxil.

The study was registered on https://classic.clinicaltrials.gov/ (registration no.: NCT04729764).

## INTRODUCTION

Influenza, commonly known as the flu, is an acute respiratory infection caused by the influenza virus. Symptoms range from mild to severe, with severe cases potentially requiring hospitalization and may even result in death. According to estimates by the Centers for Disease Control and Prevention in 2018, influenza causes approximately 290,000 to 640,000 deaths worldwide each year (1). Consequently, the prevention and treatment of influenza have become a critical public health issue.

The influenza virus can be divided into three types: A, B, and C. Type A primarily causes widespread pandemics, type B mainly leads to localized outbreaks, and type C is less commonly associated with epidemics (2). The influenza virus is highly mutable, undergoing minor antigenic variations (antigenic drift) every 23 years, and major antigenic shifts that result in new strains and large-scale pandemics occur approximately every decade (3).

The treatment of influenza primarily relies on antiviral drugs. The main types of drugs currently used to prevent and treat influenza are M2 ion channel inhibitors and neuraminidase inhibitors (NA inhibitors), with amantadine representing the former and oseltamivir the latter (4). Due to widespread resistance and significant side effects, M2 ion channel inhibitors are not recommended for clinical use, and NA inhibitors are the mainstay of treatment (4, 5). The mechanism of action of NA inhibitors is to inhibit the budding and release of the influenza virus, thereby suppressing viral spread (6). However, they do not inhibit viral replication, and it is recommended to use oseltamivir within the first 36 h of infection. With oseltamivir having been on the market for 20 years, resistance issues have also emerged (6). Therefore, there is an urgent need to develop new antiviral drugs with novel mechanisms of action that can inhibit viral replication.

In the life cycle of the influenza virus, the transcription and replication of RNA (ribonucleic acid) fragments are a key step, with the RNA polymerase playing a crucial role in the transcription and replication of the virus (7). The influenza virus RNA polymerase consists of three subunits: polymerase acidic protein (PA), polymerase basic protein 1 (PB1), and polymerase basic protein 2 (PB2) (7). The PB2 subunit is responsible for recognizing and binding to the “cap structure” of the host mRNA (messenger ribonucleic acid) precursor. The PA subunit has endonuclease activity and is responsible for cleaving host cell mRNA to serve as a primer for the initiation of viral RNA replication, and the PB1 subunit is mainly responsible for the replication and transcription of viral RNA (8, 9). The PA subunit plays a key role in the replication and proliferation of the influenza virus (8). It is highly conserved during viral mutations, making it an ideal target for new antiviral drugs (10).

Baloxavir marboxil is the first drug targeting the PA subunit, showing sound therapeutic effects against both type A and type B influenza (11). It was approved for marketing in Japan in February 2018 and by the U.S. FDA in October 2018 (11, 12). Currently, there are no drugs targeting the PA subunit available on the market in China.

Suraxavir marboxil (GP681), hereafter referred to as suraxavir, is a prodrug of a PA inhibitor, and its metabolite GP1707D07 selectively inhibits the cap-dependent endonuclease of the influenza virus, preventing viral replication with a novel mechanism of action against influenza viruses. *In vitro*, suraxavir demonstrated nanomolar antiviral activity against both influenza A and B viruses, with the average half-maximal inhibitory concentration (IC_50_) on the transcriptional activity of 0.6 nM. In comparison, the average IC_50_ of baloxavir was 1.8 nM. The concentration for 90% of maximal effect (EC_90_) of 6.87 ng/mL was also determined (13, 14). The phase Ⅲ study in outpatients aged 5–65 years with acute uncomplicated influenza (ClinicalTrials.gov registration: NCT05474755) revealed that GP681 was a safe and effective oral antiviral drug with high compliance and low drug resistance for uncomplicated influenza (13).

According to the relevant requirements of the “Drug Registration Management Measures (2020 Edition),” suraxavir tablets produced by Jiangxi Qingfeng Pharmaceutical Co., Ltd. need to undergo initial clinical pharmacology and human safety evaluation trials. The primary objective of these trials is to assess the tolerance of the drug in humans and its pharmacokinetic profile, providing a foundation for the formulation of dosing regimens in subsequent clinical trials.

## MATERIALS AND METHODS

The study consisted of two parts: the single ascending-dose study (SAD) was a randomized, double-blind, placebo-controlled study, and the food effect study (FE) was an open-label, 2-period crossover design study.

### Study design

#### SAD

**Fig 1:**
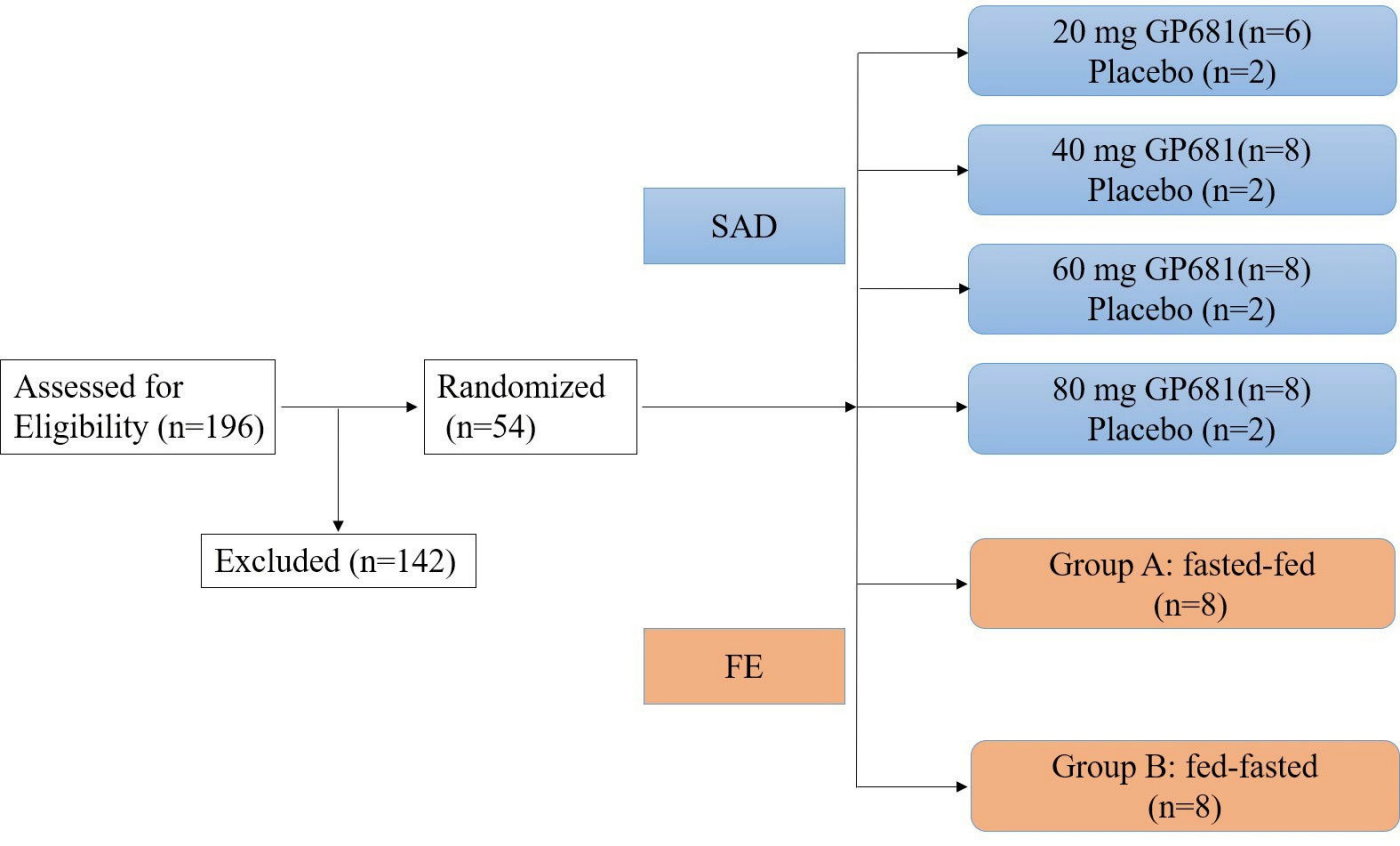
The scheme of study design.

SAD was a randomized, double-blind, placebo-controlled study, and 38 volunteers were involved in this part. Subjects were randomly assigned to one of the four groups, receiving suraxavir or placebo at doses of 20, 40, 60, and 80 mg. The initial dose group (20 mg) was designed using the “sentinel method”: two sentinel subjects were enrolled in the trial (suraxavir:placebo = 1:1), and after the two sentinel subjects completed the 72 h post-dosing safety follow-up, and it was determined that no dose-limiting toxicity had occurred, enrollment of the remaining six subjects in the dose group was initiated (suraxavir:placebo = 5:1). If the safety profile of the initial dose group is favorable, all volunteers may be enrolled at once in the following remaining dose groups (eight for suraxavir and two for placebo) (Fig. 1).

After screening, eligible volunteers were admitted to the study center 1 day before drug administration. Volunteers were administered suraxavir or placebo at the respective dose level with 240 mL of water on the morning of Day 1 in a fasted state (≥10 h). All volunteers stayed at the study center until 4 days after dosing with close medical monitoring and returned to the study center for the follow-up visits at D6, D8, and D12.

#### FE

FE was an open-label, 2-period crossover design study, 16 healthy male volunteers were randomly divided into two groups, A and B, with eight subjects in each group. Volunteers were assigned to either group A (fasting-fed) or group B (fed-fasting) according to the randomization scheme.

After screening, eligible volunteers were admitted to the study center 1 day before drug administration. On the morning of Day 1 of each period, each volunteer was administered 40 mg of suraxavir under fed or fasting conditions. Volunteers under fed conditions must have a high-fat, high-calorie breakfast (total calories: approximately 800–1,000 kcal, of which fat accounted for at least 50% of the meal) 30 min before dosing. Suraxavir was administered with 240 mL of water. Volunteers were not allowed to drink water 1 h before and 1 h after dosing, except for the water used for drug administration. Food intake was strictly controlled, and lunch and dinner were provided approximately 4 and 10 h post-dose. All the volunteers stayed at the study center until 4 days after dosing and returned to the study center for the follow-up visits at D8 and D12 in each period.

### Participants

#### Inclusion criteria

Eligible subjects were healthy Chinese males or females (the FE is restricted to males only) aged between 18 and 45 years, body weight ≥ 50 kg for males, or ≥45 kg for females, and body mass index (BMI) between 19 and 26 kg/m^2^ (included). Physical examination, vital signs, chest X-ray, 12-lead ECG, and results of laboratory tests were normal or abnormality with no clinical significance.

#### Exclusion criteria

Exclusion criteria included a medical history of central nervous system, cardiovascular system, digestive system, respiratory system, urinary system, blood system, metabolic disorders, or other diseases (e.g., history of mental illness) that are not suitable for participation in clinical trials; blood donation or blood loss ≥400 mL within 3 months before dosing; drug or alcohol addicts, or smoke more than 10 cigarettes a day; any concomitant medication within 2 weeks before screening; positive test for hepatitis B, hepatitis C, HIV, or syphilis; positive pregnancy test for females.

### Safety assessment

The safety assessment was performed through 12-lead ECGs, vital signs (body temperature, blood pressure, and pulse rate), clinical laboratory tests, physical examinations, and adverse events (AEs) monitoring throughout the study. AEs were assessed according to the National Cancer Institute Common Terminology Criteria (CTCAE, version 5.0) and were recorded and processed promptly by qualified investigators according to relevant regulations.

Dose escalation should be terminated when (i) half or more of the subjects in a dose group taking suraxavir experience any grade 2 toxicity or when one-third or more of the subjects experience any grade 3 toxicity (according to the adverse event grading criteria of CTCAE 5.0); (ii) serious adverse events related to suraxavir occur in at least one subject; and (iii) investigators find that the drug exposure level is trending toward saturation and approaching a plateau, continuation of the trial is deemed unnecessary in consultation with the sponsor.

### Biological sample collection

Blood samples were collected from the subjects’ superficial veins of the upper limbs using intravenous indwelling needles or direct lancets, and approximately 3 mL of blood was collected each time into vacuum blood collection tubes containing sodium fluoride/potassium oxalate anticoagulant. In SAD, blood samples were collected at the following 17 time points: 0 (pre-dose), 0.5, 1.0, 2.0, 3.0, 4.0, 5.0, 6.0, 8.0, 12.0, 24.0, 36.0, 48.0, 72.0, 120.0, 168.0, and 264.0 h post-dose. In FE, blood samples were collected at the following 15 time points: 0 (pre-dose), 1.0, 2.0, 3.0, 4.0, 5.0, 6.0, 8.0, 12.0, 24.0, 36.0, 48.0, 72.0, 168.0, and 264.0 h post-dose in each period. The blood samples were separated by centrifugation at 4°C, 1,500 × *g*, for 10 min. The separated plasma was divided into two centrifuge tubes (each with at least 0.7 mL of plasma) and stored frozen at −80°C until analysis.

### Bioanalytical procedures

Suraxavir is a prodrug, and it can rapidly transform into GP1707D07 in humans. GP1707D07 displays antiviral activity within the body. In the SAD and FE, both plasma concentrations of suraxavir and GP1707D07 were determined by liquid chromatography-tandem mass spectrometry (LC-MS/MS) in Frontage Laboratories (Suzhou) Co., Ltd. The samples were pretreated by the protein precipitation method. GP1707D06 was chosen as the internal standard, and the ranges of GP681 and GP1707D07 in human plasma were 0.3–300 ng/mL and 0.3–300 ng/mL, respectively. The main instruments include a Triple Quad 6500^+^ mass spectrometer (AB SCIEX, USA) and High Performance Liquid Chromatography System LC-30AD (Shimadzu, Japan).

### Pharmacokinetic assessments

Pharmacokinetic parameters were calculated using PhoenixWinNonlin 8.1 software (Pharsight, Cary, NC, USA). Concentration–time data of GP681 and GP1707D07 were analyzed by noncompartmental methods. Pharmacokinetics (PK) parameters mainly included the following: AUC from time 0 (pre-dose) to the time of the last measurable concentration (AUC_0–*t*_), AUC from time 0 (pre-dose) to infinity (AUC_0–∞_), maximum observed plasma concentration (*C*_max_), time to maximum plasma concentration (*T*_max_), terminal elimination half-life (*t*_1/2_), apparent distribution volume (Vz/F), clearance rate (CL/F), average retention time from time 0 to the time of the last measurable concentration (MRT_0–*t*_).

### Statistical analyses

Statistical analysis was performed using SAS Software version 9.4 or above (SAS Institute, Cary, NC, USA). Continuous variables were statistically described using the number of cases, mean, median, standard deviation (SD), minimum values, and maximum values. Categorical variables were described using each category’s frequency and percentage. A power model was used to investigate the correlation between PK parameters (AUC, *C*_max_) and doses. For FE, differences between the pharmacokinetic parameters of the metabolite GP1707D07 were compared between the fed (high-fat, high-calorie meals) and fasting groups using bioequivalence evaluation. The main pharmacokinetic parameters (*C*_max_, AUC_0–*t*_) were log-transformed and tested for significance by multifactorial analysis of variance (ANOVA) at the *α* = 0.05 level, with sequence, period, and treatment group (fasting and fed group) as fixed effects and subject (sequence) as a random effect in the ANOVA model, and then the geometric mean ratio (GMR) of the main pharmacokinetic parameters (fasting group/fed group) and the 90% confidence intervals (CIs) of GMR of the fasting and fed group were calculated. Nonparametric testing was used to compare the difference in *T*_max_ between the two states. If the 90% CIs were within the range of 80.0%–125.0%, and there is no statistically significant difference in *T*_max_, it can be determined that food does not affect the pharmacokinetic profile of the drug.

## RESULTS

### Demographic profile

In SAD, a total of 139 healthy subjects were screened. Thirty-eight eligible volunteers were enrolled, and all of them completed the study. In FE, a total of 57 healthy subjects were screened. Sixteen eligible volunteers were enrolled and randomly assigned 1:1 to treatment group A or group B, and all of them completed the study. The distribution of volunteers is displayed in Fig. 1. The demographic profile of all enrolled volunteers is demonstrated in Table 1.

**TABLE 1:** Demographic profile of enrolled subjects^*a*^

	Single ascending-dose study					Food effect study	
	20 mg (*N* = 6)	40 mg (*N* = 8)	60 mg (*N* = 8)	80 mg (*N* = 8)	Placebo (*N* = 8)	Group A	Group B
Age, years	28.8 (5.74)	30.4 (2.39)	30.5 (7.67)	26.5 (3.38)	32.6 (5.53)	30.6 (6.32)	28.8 (5.85)
Gender							
Male (*n* [%])	6 (100)	7 (87.5)	7 (87.5)	7 (87.5)	6 (75.0)	8 (100)	8 (100)
Female (*n* [%])	0	1 (12.5)	1 (12.5)	1 (12.5)	2 (25.0)	0	0
Height, cm	169.25 (3.03)	165.69 (5.14)	167.81 (9.39)	169.69 (9.07)	166.06 (10.77)	166.69 (5.22)	163.50 (5.35)
Weight, kg	63.17 (6.89)	58.04 (7.16)	62.46 (7.94)	66.14 (12.54)	61.64 (10.74)	62.41 (7.09)	60.50 (7.91)
BMI, kg/m^2^	22.05 (2.26)	21.06 (1.64)	22.16 (1.84)	22.80 (2.47)	22.24 (1.96)	22.40 (1.80)	22.60 (2.50)

^*a*^ Data are expressed as mean (SD), except for gender, which is shown as *n* (%).

### Safety

In SAD, six subjects (20%) taking suraxavir had nine treatment-emergent adverse events (TEAEs), and six subjects (75%) taking placebo had seven TEAEs (Table 2). All the AEs were considered to be probably unrelated to the study drug, except one case of TEAE (abnormal ECG) in the 80 mg dose group, which was reported to be related to the study drug. All AEs recovered without treatment after discontinuation of the drug.

**TABLE 2:** Treatment-emergent adverse events in SAD

	20 mg, *N* = 6	40 mg, *N* = 8	60 mg, *N* = 8	80 mg, *N* = 8	Placebo, *N* = 8
TEAE	1 (16.7)	2 (25.0)	2 (25.0)	1 (12.5)	6 (75.0)
Upper respiratory tract infection	1 (16.7)	0	1 (12.5)	0	0
Elevated blood uric acid	0	2 (25.0)	1 (12.5)	0	0
Elevated blood triglycerides	0	2 (25.0)	0	0	2 (25.0)
Urine protein detection	0	0	0	1 (12.5)	0
Intraventricular conduction block	0	0	0	1 (12.5)	0
Lower blood magnesium	0	0	0	0	2 (25.0)
Positive urine leukocytes	0	0	0	0	1 (12.5)
Elevated blood creatine phosphokinase isoenzyme	0	0	0	0	1 (12.5)
Decreased blood pressure	0	0	0	0	1 (12.5)

In FE, eight volunteers in the fasting state experienced 10 TEAEs, and four volunteers in the fasting state had four drug-related TEAEs; eight volunteers in the fed state experienced 13 TEAEs, and three volunteers in the fed state had three drug-related TEAEs (Table 3). There is no significant difference in the incidence of AEs between fasting and fed states. Except for two cases of grade 2 AEs (one case of elevated triglycerides and one case of elevated creatine kinase) under fed conditions, the severity of all other AEs is level 1. All AEs recovered without treatment after discontinuation of suraxavir.

**TABLE 3:** Treatment‐emergent adverse events in FE

	Fasting, *N* = 16	Fed, *N* = 16
TEAE	8 (50.0)	8 (50.0)
Elevated blood triglycerides	5 (31.3)	5 (31.3)
Elevated blood bilirubin	2 (12.5)	0
Elevated alanine aminotransferase	1 (6.3)	1 (6.3)
Elevated blood pressure	1 (6.3)	0
Positive urine leukocytes	0	1 (6.3)
Positive urine erythrocytes	0	1 (6.3)
Intraventricular conduction block	0	1 (6.3)
Elevated blood creatine phosphokinase	0	1 (6.3)
Elevated blood uric acid	0	1 (6.3)
Increased total bile acids	0	1 (6.3)
Headache	1 (6.3)	0

### Pharmacokinetic properties

**Fig 2:**
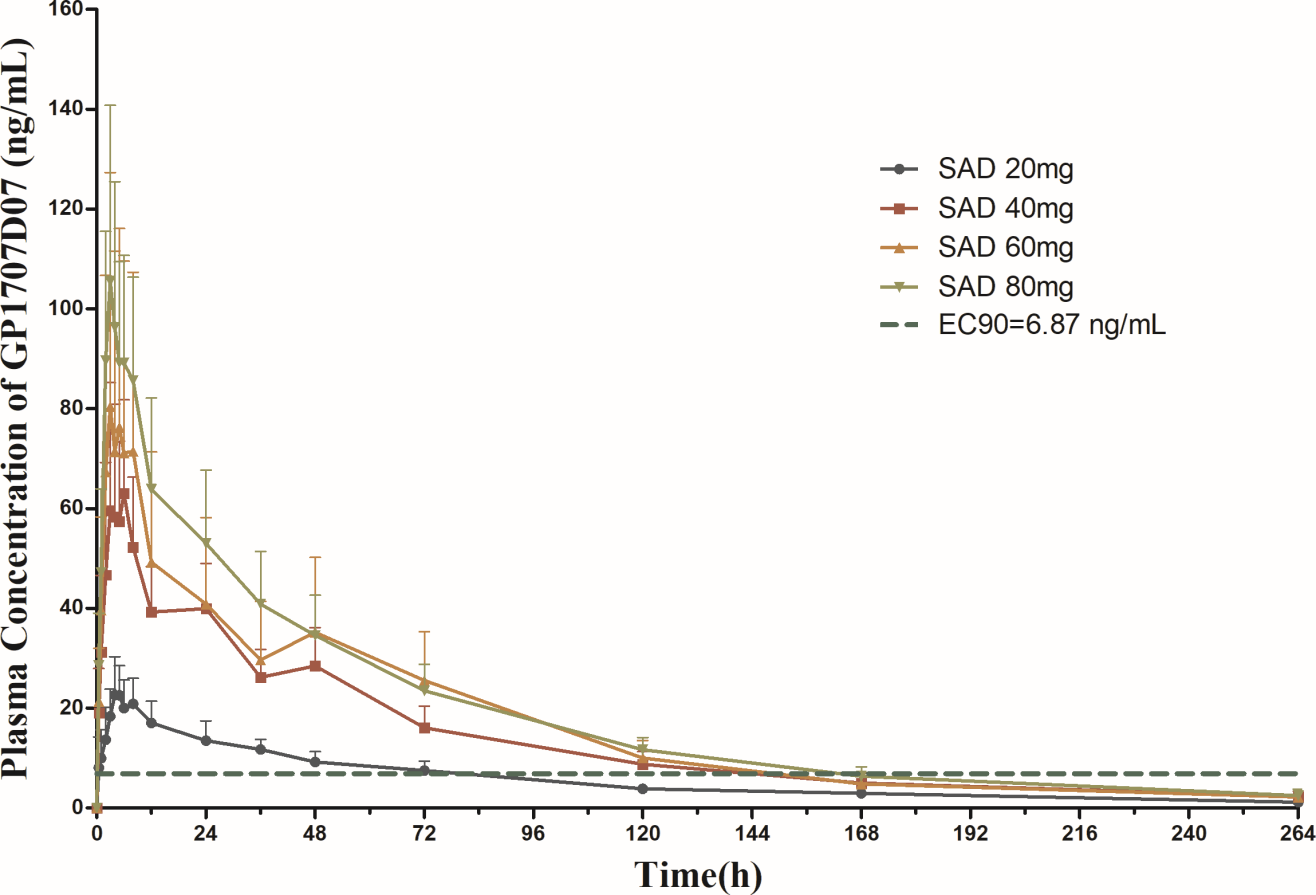
Mean plasma *c*–*t* curves of GP1707D07 in SAD.

In SAD, only five blood samples detected suraxavir, and the concentration was slightly higher than the lower limit of quantification (0.30 ng/mL), while GP1707D07 concentration increased with increasing suraxavir dose. These results indicate that suraxavir was rapidly and widely converted into its active metabolite GP1707D07 after oral administration. Thus, only the metabolite GP1707D07 was analyzed for blood concentration and pharmacokinetic parameters in this study. The primary plasma PK parameters of GP1707D07 in each dose group after a single dose of suraxavir are summarized in Table 4, and the mean plasma drug concentration–time curves are shown in Fig. 2. The results indicate that, following a single dose of suraxavir tablets at 20 mg, the average plasma concentration of metabolite GP1707D07 remains above 6.87 ng/mL (this concentration is the target blood concentration at which clinical effects are intended to be achieved and is converted to the *in vivo* drug concentration in adults using the GP681 *in vitro* antiviral-positive EC_90_ [maximum limit]) for 72 h. Correspondingly, after administering suraxavir tablets at doses of 40, 60 , or 80 mg once, the average plasma concentration of GP1707D07 persists above 6.87 ng/mL for approximately 136 h.

**TABLE 4:** Main plasma PK parameters in SAD^*a*^^,^^*b*^

Pharmacokinetic parameters	20 mg (*N* = 6)	40 mg (*N* = 8)	60 mg (*N* = 8)	80 mg (*N* = 8)
*C*_max_ (ng/mL)	23.6345 ± 6.9340	68.8669 ± 21.7872	84.5190 ± 44.4983	109.9923 ± 30.8582
*C*_24h_ (ng/mL)	13.4818 ± 3.9573	39.9186 ± 9.0409	40.8339 ± 17.3316	52.9981 ± 14.7075
*C*_72h_ (ng/mL)	7.4857 ± 1.8360	16.0573 ± 4.3543	25.5686 ± 9.7496	23.5118 ± 5.2416
AUC_0–*t*_ (h ng/mL)	1,509.625 ± 329.7046	3,595.469 ± 761.1730	4,384.829 ± 1,690.2501	5,067.380 ± 978.7640
AUC_0–24h_ (h ng/mL)	398.881 ± 100.8378	1,059.963 ± 254.1222	1,295.971 ± 621.2855	1,649.789 ± 391.4234
AUC_0–72h_ (h ng/mL)	876.695 ± 188.0006	2,318.226 ± 510.8764	2,837.817 ± 1,205.4054	3,362.445 ± 748.8872
AUC_0–∞_ (h ng/mL)	1,639.626 ± 362.1362	3,867.566 ± 805.5856	4,572.367 ± 1,727.5357	5,290.551 ± 1,036.6601
*T*_max_ (h)	4.500 (4.0, 8.0)	4.510 (3.0, 6.0)	3.000 (2.0, 8.0)	3.000 (2.0, 5.0)
*t*_1/2_ (h)	75.900 ± 12.8972	74.507 ± 25.7250	59.823 ± 8.4473	58.091 ± 10.7706
MRT_last_ (h)	73.738 ± 4.7318	65.528 ± 8.8701	64.066 ± 8.9361	61.202 ± 7.6512
CL/F (L/h)	12.731 ± 2.9334	10.681 ± 1.9026	15.667 ± 8.6060	15.674 ± 3.2594
Vz/F (L)	1,389.053 ± 388.7828	1,149.236 ± 477.7799	1,370.772 ± 771.8377	1,298.790 ± 267.4034
Kel (h^−1^)	0.0094 ± 0.0016	0.0103 ± 0.0034	0.0118 ± 0.0014	0.0122 ± 0.0020

^*a*^ Data are expressed as mean (SD), except for *T*_max_, which is shown as median (min, max).

^*b*^ *C*_max_, maximum observed plasma concentration; *C*_24h_, plasma concentration 24 h after dosing; *C*_72h_, plasma concentration 72 h after dosing; AUC_0*–t*_, the area under the concentration–time curve from time zero to the time of the last measurable concentration; AUC_0–24h_, the area under the concentration–time curve from time zero to time 24 h; AUC_0–72h_, the area under the concentration–time curve from time zero to time 72 h; AUC_0–∞,_ the area under the concentration–time curve from time zero to infinity; *T*_max_, time to maximum plasma concentration; *t*_1/2_, terminal elimination half-life; MRT_last_, average retention time from zero to infinity; CL/F, clearance rate; Vz/F (L), apparent distribution volume; Kel, elimination rate constant.

**Fig 3:**
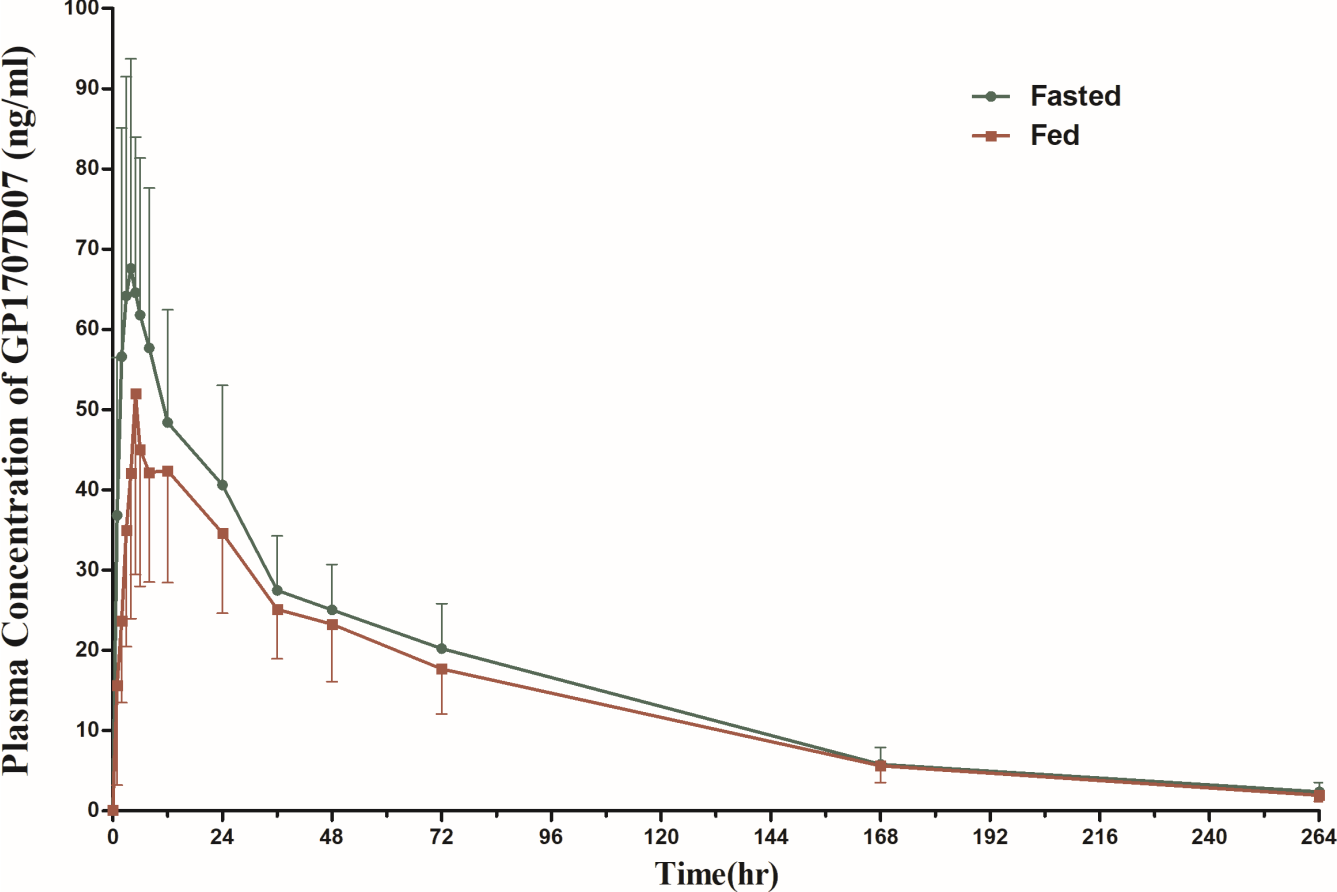
Mean plasma *c*–*t* curves of GP1707D07 in FE.

In FE, only one blood sample detected suraxavir, and the concentration was slightly higher than the lower limit of quantification (0.30 ng/mL). The mean blood concentration of GP1707D07 increased rapidly after oral administration of a single dose of GP681 in both the fasted and fed groups. The *T*_max_ of GP1707D07 under the fasted and fed conditions was 3.5 and 5 h, respectively. A high-fat, high-calorie meal reduces *C*_max_ by approximately 19% and AUC_0–∞_ by approximately 15% compared to those in the fasting condition. The primary plasma PK parameters of GP1707D07 in the fasted and fed conditions after a single dose of suraxavir are summarized in Table 5, and the mean plasma drug concentration–time curves are shown in Fig. 3.

**TABLE 5:** Main plasma PK parameters in FE^*a*^^,^^*b*^

Pharmacokinetic parameters	Fasting (*N* = 16)	Fed (*N* = 16)
*C*_max_ (ng/mL)	73.9263 ± 26.0668	59.7846 ± 18.3175
*C*_24h_ (ng/mL)	40.6127 ± 12.4344	34.6095 ± 9.9946
AUC_0–*t*_ (h ng/mL)	4,096.069 ± 1,017.7748	3,531.313 ± 920.3926
AUC_0–24h_ (h ng/mL)	1,186.523 ± 368.9721	909.474 ± 196.8328
AUC_0–∞_ (h ng/mL)	4,327.816 ± 1,050.3557	3,703.653 ± 972.7299
*T*_max_ (h)	3.5 (2, 8)	5.0 (3, 12)
*t*_1/2_ (h)	62.235 ± 13.2211	59.250 ± 7.6860
MRT_last_ (h)	64.727 ± 9.0765	66.834 ± 6.9822
CL/F (L/h)	9.885 ± 2.9309	11.623 ± 3.4842
Vz/F (L)	886.507 ± 321.0009	983.230 ± 274.1432
Kel (h^−1^)	0.0116 ± 0.0023	0.0119 ± 0.0014
*F* (%)	120.091 ± 34.7642	

^*a*^ Data are expressed as mean (SD), except for T_max_, which is shown as median (min, max).

^*b*^ *C*_max_, maximum observed plasma concentration; *C*_24h_, plasma concentration 24 h after dosing; AUC_0–*t*_, the area under the concentration–time curve from time zero to the time of the last measurable concentration; AUC_0–24h_, the area under the concentration–time curve from time zero to time 24 h; AUC_0–∞,_ the area under the concentration–time curve from time zero to infinity; *T*_max_, time to maximum plasma concentration; *t*_1/2_, terminal elimination half-life; MRT_last_, average retention time from zero to infinity; CL/F, clearance rate; Vz/F (L), apparent distribution volume; Kel, elimination rate constant; *F*, relative bioavailability.

## DISCUSSION

In this first-in-human phase I study of suraxavir, a single dose of suraxavir (20, 40, 60, and 80 mg) was well tolerated in healthy Chinese subjects, and all AEs were mild and transient. Furthermore, after a single oral dosing, the prodrug suraxavir was rapidly metabolized to the active metabolite GP1707D07. The pharmacokinetics of GP1707D07 were linear and had a long elimination half-life. In addition, the FE was conducted to evaluate the food effect on the pharmacokinetics of suraxavir tablets.

In the single-dose escalation study, AEs were probably unrelated to suraxavir except for one case of AE (intraventricular conduction block) in the 80 mg dose group. Both subjects suffering from upper respiratory infections had reported triggers such as cold weather or rain and were consequently deemed by the study physician to be unrelated to suraxavir. In the FE, there was no significant difference in the incidence of AEs between the fasted state and the high-fat, high-calorie meal state, suggesting that administration of the drug in the fed or fasted state does not affect the drug’s safety. All AEs were transient abnormalities that recovered without treatment after discontinuation of the drug. The AEs observed in this study are basically consistent with those of baloxavir marboxil (15).

Except for five samples in the SAD and one sample in the FE, the blood concentration of suraxavir was slightly higher than the lower limit of quantification (LLOQ, 0.30 ng/mL). The blood concentration of GP681 in all other samples was lower than the LLOQ, indicating that suraxavir was almost entirely rapidly hydrolyzed and converted into its active metabolite GP1707D07 *in vivo* after oral administration.

The study results showed that the blood concentration of GP1707D07 increased proportionally with the increase of the dosage of suraxavir when it was administered within the range of 20–80 mg. The trend of blood concentration over time was basically the same among the dosage groups, which indicated that it was positively correlated with the dosage of suraxavir tablets. The mean time to reach *C*_max_ (*T*_max_) for the active metabolite GP1707D07 was approximately 4.0 h (3.0–4.5 h). The average plasma concentration of the metabolite GP1707D07 was higher than the target blood concentration of suraxavir at 72 h after single-dose oral administration of 20 mg of suraxavir tablets and at about 136 h after single-dose oral administration of 40, 60, and 80 mg of suraxavir tablets, which suggests that an effective anti-influenza viral effect can be achieved by oral administration of suraxavir tablets at a dose of 20 mg or higher. In the 20–80 mg dose range, adequate anti-influenza viral blood concentrations can be maintained for approximately 72–136 h.

A total of 48 healthy subjects (male and female) were planned to be randomly enrolled in the original five dose groups(20, 40, 60, 80, and 120 mg) of the single administration dose escalation study. Results from the 20–80 mg dose groups showed that after a single oral administration of suraxavir tablets at doses of 20 mg and above, the *C*_24h_ blood concentrations in healthy subjects in all dose groups had exceeded the target efficacy blood concentration (≥6.87 ng/mL). After evaluating the dose and resistance risk of the same target drug by the sponsor and the investigators, it was considered that 80 mg or less doses were sufficient to meet clinical medication needs. Therefore, the trial of the 120 mg dose group was subsequently terminated, and the maximum dose of 80 mg was considered the maximum tolerated dose.

The results of the FE showed that the geometric mean ratios and their 90% CIs of the pharmacokinetic parameters *C*_max_, AUC_0–*t*_, and AUC_0–∞_ of GP1707D07 in the fasted state versus the state of having a high-fat, high-calorie meal were 120.99% (105.79%, 138.38%), 116.22% (106.21%, 127.18%), 117.29% (107.19%, 128.34%), and the upper limit of the 90% CIs of all three pharmacokinetic parameters exceeded the upper limit of the acceptable range of 80.00%–125.00%. The median *T*_max_ of GP1707D07 in the fasting state was about 3.5 h and was slightly delayed to 5.0 h in the fed state. A high-fat, high-calorie meal reduces *C*_max_ by approximately 19% and AUC_0–∞_ by approximately 15% compared to those in the fasting condition. Therefore, food (high-fat, high-calorie meals) may slightly reduce the rate and extent of drug absorption.

Based on these results, suraxavir was safe and generally well tolerated. In conjunction with the consideration of the target blood concentration of 6.87 ng/mL for the intended clinical onset of action of the drug, these findings suggest that subsequent clinical trials may be conducted with appropriate oral doses in the dose range of 20–80 mg. In a phase Ⅲ trial, a single dose of suraxavir was found to effectively reduce the time to alleviation of influenza symptoms and safely reduce influenza viral load in uncomplicated influenza patients aged 5–65 years (12). On 27 March 2025, the National Medical Products Administration approved suraxavir for the treatment of uncomplicated influenza A and B in previously healthy adolescents aged 12 and above and adults (16).

### Conclusions

Suraxavir was well tolerated in healthy Chinese participants, and the *C*_24h_ blood concentration in healthy Chinese participants is sufficient to meet the clinical needs within a single-dose range of 20–80 mg. Based on the safety and pharmacokinetic data, it is recommended to use 20–80 mg promptly for further clinical development.

## Data Availability

The original contributions presented in the study are included in the article/supplementary material, further inquiries can be directed to the corresponding authors.
